# Modified Arthroscopic Suture Fixation of Posterior Cruciate Ligament Tibial Avulsion Fracture in the Setting of Multiligament Knee Injury in Teenager

**DOI:** 10.1155/2021/3626276

**Published:** 2021-07-17

**Authors:** Miguel Quesado, Ana Sofia Esteves, Nuno Vieira da Silva, Tiago Costa, Nuno Ferreira, Hélder Nogueira

**Affiliations:** Serviço de Ortopedia e Traumatologia, Centro Hospitalar do Tâmega e Sousa, Penafiel, Portugal

## Abstract

The posterior cruciate ligament (PCL) avulsion fracture is a rare injury and occurs mainly in young patients. The development of arthroscopic techniques and fixation methods has improved the treatment of this entity. This report describes a modified arthroscopic suture fixation of a small tibial avulsion fracture of the PCL. A 17-year-old male, injured in a motorcycle crash, was admitted to the Emergency Department and diagnosed with left knee PCL tibial avulsion fracture, lateral collateral ligament (LCL) femoral avulsion fracture, and patella fracture. The PCL was fixed arthroscopically using a Knee Scorpion and two SutureTapes (Arthrex, Munich-Germany) through of an interlaced configuration at the base of the fragment using a transseptal approach and fixed distally over a cortical button on the anterior cortex. The LCL was repaired with two cannulated screws by a percutaneous approach. At 1 year of follow-up, the fragment was healed with tibiofemoral congruence, and the knee was stable with complete range of motion. The Tegner Lysholm Knee Scoring Scale (TLKSS) was 92.

## 1. Introduction

PCL has a central role in knee biomechanics and stability. It represents the center of rotation of the knee, and it is the main structure to resist to posterior tibial translation [1]. Its damage impairs knee biomechanics with increased contact pressures in medial tibiofemoral and patellofemoral compartments and consequently progressive degenerative changes [2]. Avulsion fractures of PCL are more frequent in the younger age group and associated to high energy trauma [1, 3]. Reduction and fixation of the fragment assisted by arthroscopy favor minimal scarring, less tissue trauma, and early rehabilitation than open approaches, and it is actually indicated for PCL injuries with displacement and posterior instability [2, 4, 5]. Some procedures for suture fixation using different materials were described by several authors in the last 20 years. This report describes a new suture placement configuration for a small fragment and comminuted tibial PCL avulsion fracture that allows anatomical reduction and great compression at PCL footprint in one case of multiligament knee injury.

## 2. Case Presentation

**Figure 1:**
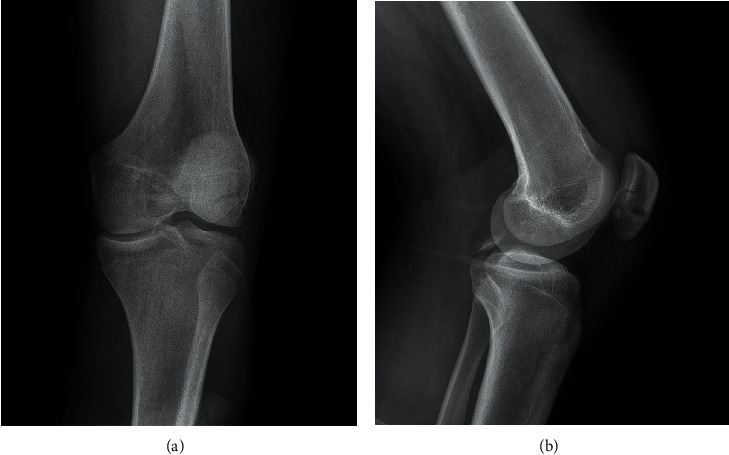
Coronal (a) and sagittal (b) X-ray on admission to the Emergency Department.

**Figure 2:**
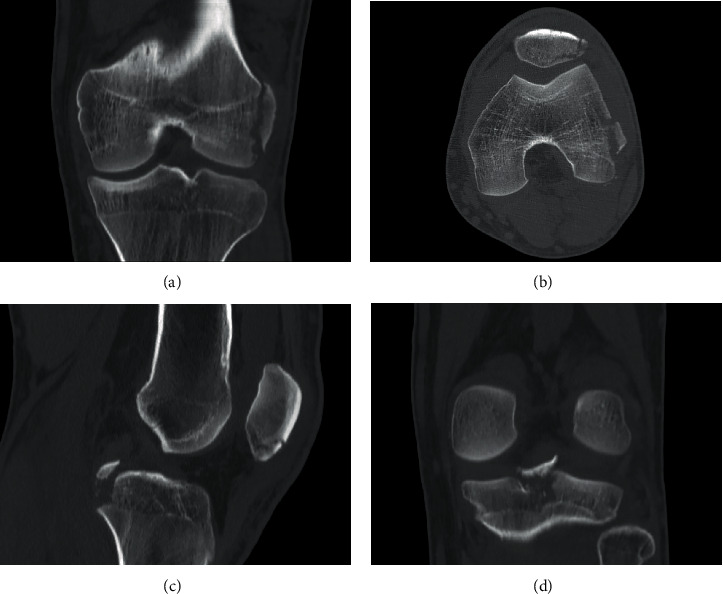
Coronal (a, d), axial (b), and sagittal (c) CT scan on admission to the Emergency Department.

**Figure 3:**
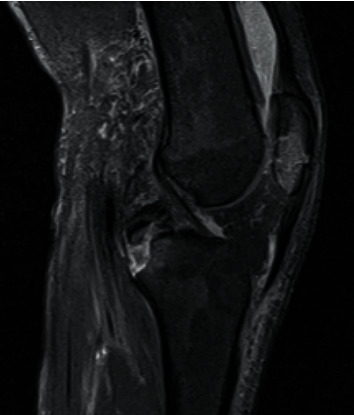
MRI (fat-saturated T2-weighted) lateral view showing a displaced avulsion fracture of PCL at tibial attachment.

A 17-year-old male was injured in a fall while riding a motorcycle. He presented left knee pain, swelling and partial active mobilities. The extensor mechanism was intact, and he had no neurovascular deficits. At clinical examination, he presented a positive posterior drawer test and positive varus stress test with a soft endpoint. The radiograph (X-ray) and computed tomography scan (CT scan) showed a displaced avulsion fracture of the PCL tibial attachment, a displaced avulsion fracture of the LCL femoral attachment and a comminuted fracture of the inferior pole of the patella (Figures 1 and 2). The PCL tibial avulsion fracture was better characterized by magnetic resonance imaging (MRI) (Figure 3). The limb was immobilized with a cast and the patient was proposed for surgical intervention.

## 3. Surgical Technique

### 3.1. Patient Positioning

The patient was positioned supine on the operative table under general anesthesia with the leg flexed at 90°. A tourniquet was used in surgery as there are no contraindications.

### 3.2. Portals

**Figure 4:**
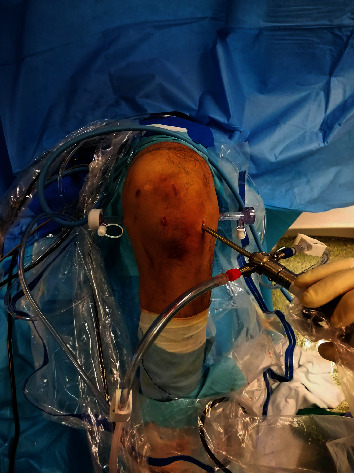
Arthroscopic portals used during the procedure (two standard anterior and two accessory posterior).

Two standard arthroscopic portals (anteromedial and anterolateral) and two accessory arthroscopic portals (posteromedial and posterolateral) were used (Figure 4).

### 3.3. Diagnostic Arthroscopy

The joint was examined through the anteromedial and anterolateral portals: an ACL pseudolaxity was observed; there were no meniscal or cartilage injuries. The arthroscope inserted from the anterolateral portal was advanced through the transcondylar notch to the posteromedial compartment: a tibial avulsion fracture of PCL with a small and comminuted fragment was observed. Under direct visualization, a spinal needle helps to identify the location and direction of the posteromedial portal. The arthroscope inserted from the anteromedial portal was advanced at the same manner to the posterolateral compartment. Under direct visualization, a spinal needle helps to identify the location and direction of the posterolateral portal. Plastic cannulas were kept in both posterior portals during the procedure. The transeptal approach was performed to expose, debride and manipulate accurately the PCL footprint. A Switching Stick was advanced from the posterolateral portal through the posterior septum. Careful orientation and direct visualization were essential in order to not compromise the posterior neurovascular structures. Following, a shaver from posteromedial portal facing anterior was advanced from medial to lateral. At this moment, careful dissection of the PCL footprint and cleaning of the scar tissue were performed with the arthroscope in posterior portals.

### 3.4. Fixation of PCL Tibial Attachment

**Figure 5:**
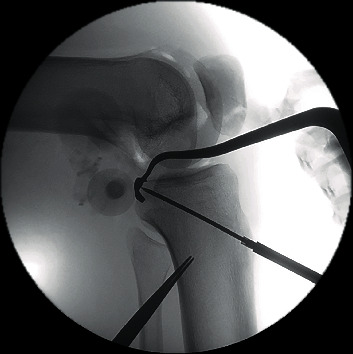
Insertion of K-wires to the PCL tibial footprint controlled by fluoroscopy.

**Figure 6:**
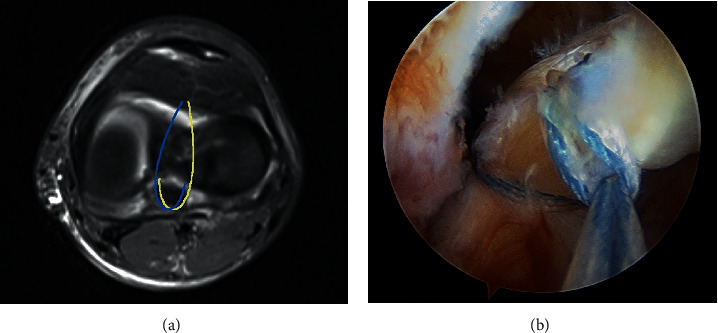
MRI (T2-weighted) axial view - schematic representation of each suture (yellow and blue lines) crossing the posterior part of the base of the PCL and exiting the contralateral tunnel (a). Arthroscopic posteromedial view - interlaced suture configuration for PCL avulsion fracture (b).

The PCL footprint on the tibia was identified by posteromedial portal. The center was raw using a curved rasp by the posterolateral portal. Blood clots, free bony fragments, and soft tissue interposed were debrided using the shaver until the avulsed fragment and tibial bony bed could be clearly released from the posterior capsule. A 2.5 cm longitudinal incision was made just medial to the tibial tubercule, and two parallel 2.5 mm K-wires were inserted through the tibial PCL guide to the medial and lateral borders of the tibial footprint, respectively (Figure 5). A Knee Scorpion loaded with SutureTape was inserted through the posteromedial portal, and a cinch stitch was created in the medial PCL tibial footprint. Similarly, the process was repeated through the posterolateral portal. At this time, the PCL footprint presented two cinch stitches at its medial and lateral portions. A 1.0 mm shuttle suture was inserted via the medial bone tunnel. The procedure was repeated for the lateral tunnel. Each suture crossed the posterior part of the ligament, passed the loop, and pulled to the outside through the contralateral tunnel, creating an interlaced suture configuration (Figure 6). Tensioning was performed with the knee at 90° of flexion, applying an anterior force to the proximal tibia and maintaining tension in both sutures. Arthroscopic reduction was confirmed by the relationship of the condyles and meniscus and the restoration of the articular step-off. The threads were fixed on the tibia using a cortical button fixation.

### 3.5. Fixation of LCL Femoral Attachment

**Figure 7:**
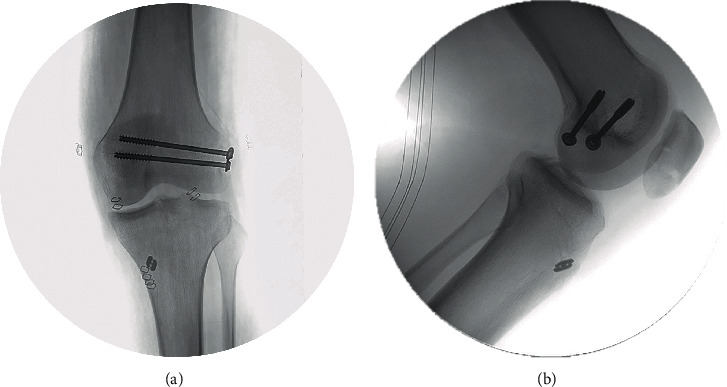
Coronal (a) and sagittal (b) intraoperative X-ray showing the final reduction and fixation of PCL and LCL avulsion fractures.

The leg was flexed at 30° with valgus force. Closed reduction of the fragment of LCL attachment at the lateral femoral condyle was performed under fluoroscopic control. Two percutaneous screws (4.0 mm) were inserted and fixed the fragment (Figure 7).

### 3.6. Postoperative Rehabilitation

**Figure 8:**
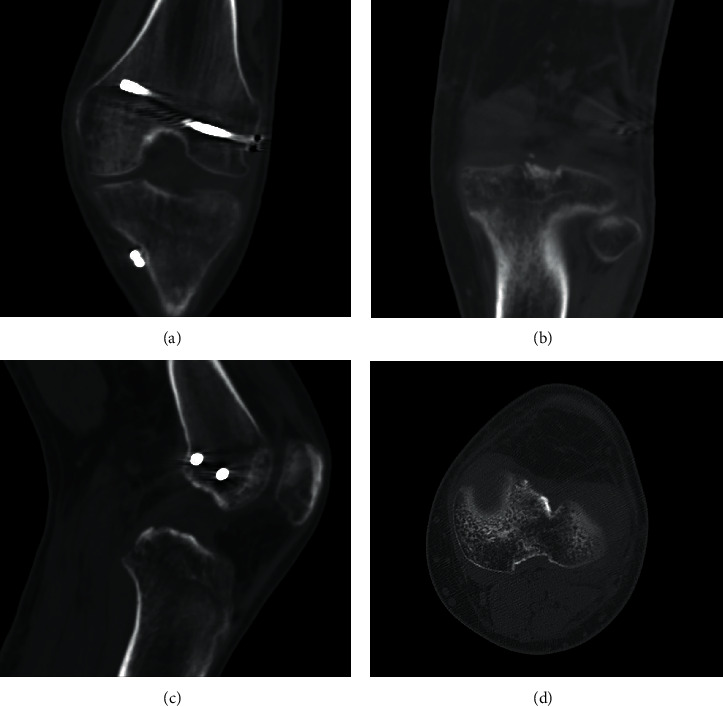
Coronal (a, b), sagittal (c), and axial (d) control CT scan after 3 months postoperative with evidence of fracture healing.

The limb was immobilized in extension with a cast during 2 weeks. Then, the patient started isometric exercises and passive mobilization with a PCL dynamic brace. Progressive weight bearing and active full range of motion were performed after 2 months postoperative. Three months after surgery, the avulsion fractures of PCL and LCL and the patella fracture were healed (Figure 8). The patient returned to sports 6 months later without limitations or sequelae. After 1 year of follow-up, the TLKSS was 92.

## 4. Discussion

PCL is the primary restraint to posterior tibial translation and the secondary restraint to external rotation of the knee joint [1]. Its injury varies from 3 to 23% of all knee injuries, and it is more frequent in the younger age group [3, 6]. It is commonly seen as part of multiligament knee injuries and associated to an increase of road traffic accidents and sports injuries [1, 3, 7]. The true incidence of multiligament knee injuries in the skeletally immature population is unknown owing the difficulty to promptly identify it at admission, and there is no consensus regarding the optimal management [6]. In young patients, these injuries mainly manifest as an avulsion fracture due to the strength of the ligamentous structures and weakness of the bone and physis; in contrast, adults are more likely to experience ligamentous failure within the substance of the ligament [7]. PCL rupture can occur at its mid substance, femoral, or tibial attachment, with or without bony avulsion [1, 6].

Conservative treatment is indicated for avulsion fractures with a mild displacement, whereas surgery should be performed in cases of displaced avulsion fracture of PCL >5 mm and injuries with >10 mm of posterior instability [1, 5]. Until recently, the posterior open approach has commonly used but it was thought that it would entail more risks and morbidity. This approach should be reserved for cases of delayed diagnosis because of possible nonunion, which require an adequate and easier exposure of the PCL and tibial avulsion site [8]. Song et al. [4] refers in a meta-analysis that all studies on the posterior approach and arthroscopic fixation for displaced PCL avulsion fractures have satisfactory results, despite significant heterogeneity among studies with various techniques, fixation devices, and sizes of fracture fragments and follow-up periods. However, the development of the arthroscopic techniques and minimally invasive approaches provides a faster recovery and gives the chance of diagnosis and treatment of concomitant intraarticular injuries [4, 5]. Martinez-Moreno and Blanco-Blanco [9] performed the first arthroscopically assisted fixation of PCL avulsion fractures in cadaveric knees in 1988. The first report of an arthroscopic technique in vivo for PCL fixation using 3 canulated screws was described in 1995 by Littlejohn and Geissler [10]. In the last 20 years, some authors have been enhancing different types of fixation, including cannulated screws, wires or sutures, and these all proven to be effective [3]. The pullout procedure can be a solution to fix small and comminuted fragments to the tibial footprint that would be difficult to reduce through another way. The new configuration described in this case for the PCL avulsion fracture allowed to embrace the ligament and to create a high strength fixation with a large area for support. Despite many authors do not consider the use of the posterolateral portal, the association of posterior portals through a transseptal approach offers a direct view to posterior knee, allowing a better and more secure way for debridement and repair of the structures around the posterior capsule [5].

High rates of concomitant ligament or meniscus lesions are seen in PCL injuries, warranting a thorough knee preevaluation, including clinical examination and imaging studies. Fanelli and Edson [11] reported 96.5% of PCL injuries occurring in combination with other ligaments. In this case, the size of the LCL avulsion fracture allowed an effective osteosynthesis and healing with percutaneous screws, restoring the normal function of the knee. An early surgical repair was also essential to avoid arthrofibrosis and loss of the knee motion, being this the most common long-term complication observed after multiligament knee injury [6, 7].

This surgical procedure is simple and less invasive than previous methods, requiring no additional arthroscopic devices. It allows the repair of the PCL lenght, union of the fragments, and an early range of motion with minimal morbidity.
